# What the Dynamic Systems Approach Can Offer for Understanding Development: An Example of Mid-childhood Reaching

**DOI:** 10.3389/fpsyg.2017.01774

**Published:** 2017-10-10

**Authors:** Laura Golenia, Marina M. Schoemaker, Egbert Otten, Leonora J. Mouton, Raoul M. Bongers

**Affiliations:** Center for Human Movement Sciences, University Medical Center Groningen, University of Groningen, Groningen, Netherlands

**Keywords:** motor development, mid-childhood, dynamic systems approach, goal-directed reaching, developmental trends, action-perception

## Abstract

The Dynamic Systems Approach (DSA) to development has been shown to be a promising theory to understand developmental changes. In this perspective, we use the example of mid-childhood (6- to 10-years of age) reaching to show how using the DSA can advance the understanding of development. Mid-childhood is an important developmental period that has often been overshadowed by the focus on the acquisition of reaching during infancy. This underrepresentation of mid-childhood studies is unjustified, as earlier studies showed that important developmental changes in mid-childhood reaching occur that refine the skill of reaching. We review these studies here for the first time and show that different studies revealed different developmental trends, such as non-monotonic and linear trends, for variables such as movement time and accuracy at target. Unfortunately, proposed explanations for these developmental changes have been tailored to individual studies, limiting their scope. Also, explanations were focused on a single component or process in the system that supposedly causes developmental changes. Here, we propose that the DSA can offer an overarching explanation for developmental changes in this research field. According to the DSA, motor behavior emerges from interactions of multiple components entailed by the person, environment, and task. Changes in all these components can potentially contribute to the emerging behavior. We show how the principles of change of the DSA can be used as an overarching framework by applying these principles not only to development, but also the behavior itself. This underlines its applicability to other fields of development.

## Introduction

An increasing popular view in developmental psychology that has been used to understand how developmental changes emerge is the Dynamic Systems Approach (DSA) (e.g., Thelen and Smith, 1994; Lewis, 2011; Molenaar et al., 2014; Blumberg et al., 2017). The hallmark of the DSA is its emphasis on all components of the system including environment and task. The DSA has provided lasting changes in understanding development in a variety of fields (e.g., Thelen et al., 2001; van Geert, 2011; Kahrs and Lockman, 2014; Roche et al., 2016; Bateson, 2017), in particular that of infant reaching (Clifton et al., 1993; Thelen et al., 1993, 1996; Corbetta and Snapp-Childs, 2009). In general, first reaching movements to a target emerge around 3 months (von Hofsten, 1991), while by 6 months, infants develop straighter and smoother reaches (Berthier and Keen, 2006). An important contribution of the DSA is that the intrinsic dynamics of the system affect this development (Thelen et al., 1996). For example, Thelen et al. (1996) showed that infants’ activity prior to reaching and infants’ arm movement speed influenced how infants learn to reach.

**FIGURE 1:**
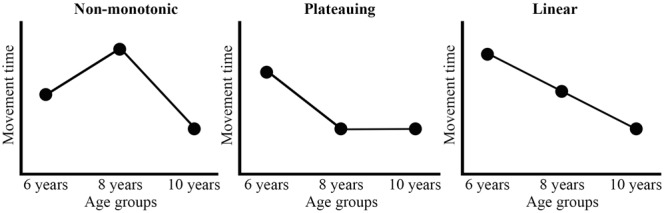
Schematic representation of different developmental trends found in reaching studies examining mid-childhood development. The variable movement time is functioning as an example. The non-monotonic developmental trend is sketched on the **Left**, the plateauing developmental trend in the **Middle** and the linear developmental trend is sketched on the **Right**. Note that the trends are in the opposite direction (a decrease is an increase and an increase is a decrease) for the variable accuracy.

In contrast to infant reaching, the DSA is underrepresented in studies of mid-childhood (6- to 10-years of age) reaching. This field is exemplified here to show how the DSA can be used as an explanatory framework to advance understanding of development. During mid-childhood reaching skills are further refined (i.e., reaches become faster and more accurate), marking this as an important developmental period which has received limited attention. Our review of the literature revealed that developmental trends differ among studies, as shown in **Figure 1** and explained in more detail later (e.g., Hay, 1979; Chicoine et al., 1992; Ferrel et al., 2001; Van Braeckel et al., 2007). Moreover, proposed explanations were tailored to trends revealed in individual studies, limiting their scope. The DSA is able to explain different developmental trends within one skill, as it focuses on all contributing components. Importantly, the components contributing to reaching are still undergoing developmental changes during mid-childhood. For example, joint coordination changes (Schneiberg et al., 2002), body proportions such as length and mass fluctuate (Malina et al., 2004), postural control accompanying reaching movements develops (van der Heide et al., 2003), and attention and executive functions improve (Olivier et al., 2003; Klimkeit et al., 2004). This shows the continuing complexity in reaching development and the need to understand developmental changes. The goal of this perspective is to offer novel ideas on how to advance the understanding of the development of mid-childhood reaching by approaching the developmental changes from the DSA. We commence with a short synopsis of the existing explanations for developmental changes in mid-childhood reaching.

## Age-Related Changes in Goal-Directed Reaching During Mid-Childhood: Short Synopsis of the Existing Studies

We focus on studies in which simple reaching movements from a start location to a target were performed. These studies have primarily focused on the performance level of the reach, quantifying spatio-temporal measures of the index finger, such as movement time or accuracy at the target. Most studies compared three age groups, usually 6-, 8-, and 10-years-olds. Based on our literature review, we differentiate developmental trends into three groups, i.e., non-monotonic (not consistently decreasing; **Figure 1**, left panel), plateauing (decrease ending in plateau; **Figure 1**, middle panel), and linear (consistently decreasing; **Figure 1**, right panel).

### Non-monotonic Developmental Trend

Several studies focusing on simple reaching movements to a target while manipulating the availability of visual feedback about the arm (vision vs. no-vision condition) observed a non-monotonic developmental trend in spatio-temporal variables (Hay, 1979; Bard et al., 1990; Hay et al., 1991; Chicoine et al., 1992; Fayt et al., 1992, 1993; Pellizzer and Hauert, 1996). A performance decrease around 8-years of age characterized this trend. For example, 6-year-old children had longer movement times than 10-year-old children, but shorter movement times than 8-year-old children (Bard et al., 1990). Interestingly, depending on the study this trend was found in the vision and no-vision condition (Pellizzer and Hauert, 1996), only in the no-vision condition (Hay, 1979; Fayt et al., 1992), or the occurrence of this trend depended on other experimental conditions, such as amplitude and direction manipulations (Bard et al., 1990; Fayt et al., 1993). Even within the same manipulation different results were found as it was the case for the amplitude and direction manipulation: for the directional error, Fayt et al. (1993) found a non-monotonic trend in both visual conditions, whereas Bard et al. (1990) found it only in the no-vision condition.

Two explanations for the non-monotonic trend were suggested in these papers. One group of authors proposed that the non-monotonic trend at the performance level reflects developmental changes of feedforward/feedback processes (Hay, 1979; Bard et al., 1990; Hay et al., 1991): 6-year-olds supposedly use feedforward processes, whereas 8-year-olds use feedback processes, while these processes would be integrated in 10-year-olds. Authors suggested that the change from feedforward to feedback control would cause the performance decrease around 8 years. Another group of authors finding the non-monotonic trend suggested that this trend reflects developmental changes in sensory integration (von Hofsten and Rösblad, 1988; Chicoine et al., 1992). They assumed that 6-year-old children rely on an intra-modal mode, in which different afferent sources (visual, proprioceptive, or tactile) are processed independently. Authors proposed that the integration of different sensory modalities would be established around the age of eight causing performance decline.

### Plateauing Developmental Trend

Studies that unexpectedly displaced the target location during the reach found a plateauing developmental trend, indicated by a performance plateau from 8-years onward (Van Braeckel et al., 2007; Wilson and Hyde, 2013). Time to correction (i.e., the time from target displacement to movement correction) decreased from 6- to 8-year-olds, but remained steady even until 12-years of age. Authors proposed that this performance improvement from 6- to 8-year-old children is the result of gaining the ability to generate an internal model (i.e., predicting the outcome of movement commands) and to integrate online sensory feedback within this model enabling rapid online correction (Wilson and Hyde, 2013). A plateauing trend was also found in a task where children had to adapt their movements to a visuomotor rotation, in which received visual feedback about the movement was rotated to a certain degree (Contreras-Vidal et al., 2005). 8-year-old children in contrast to 6-year-old children showed after-effects (more errors) in trials under non-perturbed visual feedback performed directly after a series of perturbation trials, indicating that 8-year-olds adapted to the visual perturbation. Contreras-Vidal et al. (2005) suggested that a representation (possibly related to an internal model) changes in such a way around 8 years that children can adapt to perturbed visual feedback. Note that these results are in contrast with King et al. (2009) who found after-effects in all age groups. Interestingly, they found no differences between ages in the extend of the after-effect, indicating no developmental trend for this variable.

### Linear Developmental Trend

A linear developmental trend was found in Ferrel et al. (2001), which also used a visuomotor rotation task, but focused on changes in perturbation trials instead of after-effects. Authors found a linear decrease in errors from 6- to 10-year-olds. Results were interpreted by these authors as showing changes in the nature of representations. Also studies that manipulated the availability of visual feedback found linear trends in performance measures (e.g., Olivier and Bard, 2000; Thomas et al., 2000; Takahashi et al., 2003) which is in contrast with studies described in Section “Non-monotonic Developmental Trend” which found non-monotonic developmental trends.

## Critical Reflection on Explanation of Findings

The literature overview shows that several performance measures improve over development, indicating that important fine-tuning takes place during mid-childhood that demands understanding. Reviewed studies assumed that developmental changes in performance measures follow directly from developmental changes in one single process (i.e., feedback/feedforward mechanisms, sensory integration) or component (i.e., representations) in the system (e.g., Hay, 1979; Chicoine et al., 1992; Ferrel et al., 2001). Note, we will call these approaches ‘single-cause approaches.’ The proposed cause differed across studies which might be partly due to the time of publication, spreading across four decades. Early studies were published at a time in which the information processing approach was popular, whereas more recent studies follow the computational neuroscience tradition referring to internal models and representations as explanations. In sum, over the last decades, useful and interesting explanations of developmental changes in reaching during mid-childhood have been put forward by focusing on specific single causes, fitting within the theoretical framework underlying these studies.

We, however, propose that if one wants to understand the full range and complexity of the revealed developmental trends, one should depart from the assumption that over development the same cause is responsible for all developmental changes. Our reasons for this are: first, from the literature overview it became clear that different developmental trends were found for both, different manipulations and the same manipulations (e.g., vision availability). For example, the studies of Bard et al. (1990) and Fayt et al. (1993) both used similar age-groups, manipulated vision availability and focused on amplitude and directional aspects, but they found different results. If there would be a single cause, the changes brought about by this cause should be found across manipulations. Feedback and feedforward processes, for example, play a role in all described experiments in one way or another, which would mean that the deterioration in performance following from increased usage of feedback in 8-year-olds should be seen in each experiment. As the literature overview revealed, the deterioration around 8-years is not found in all studies which makes it unlikely that there is only one cause. Second, if we follow the reasoning of ‘single-cause approaches,’ measuring one level of the system would suffice (such as the performance level) because all other levels of the system (for instance, joint angles, muscle activation patterns, or brain activation patterns) should also reflect the developmental changes of the single process or component. Studies on reaching during mid-childhood have not focused on other levels so far, however, in other behavior different developmental trends at different levels have been found (e.g., Ricken et al., 2004; Wu et al., 2009). We expect that the same is true in reaching, which argues against the reasoning of ‘single-cause approaches.’ Third, a fundamental issue that previous studies have not addressed is why these processes or components should develop in the way the authors propose. We think that this is an essential question which will be difficult to answer, hampering full understanding of the developmental changes.

## Dynamic Systems Approach to Development

We propose that the DSA (Turvey and Fitzpatrick, 1993; Thelen and Smith, 1994; Lewis, 2011; Spencer et al., 2011; Newell and Liu, 2012; Molenaar et al., 2014; Endedijk et al., 2015) can offer an explanation for the full complexity of the development of reaching during mid-childhood. In contrast with ‘single-cause approaches,’ the DSA takes all components of the system into account. Importantly, the system is not confined to the body, but includes the full action-perception cycle. Automatically, this means that the environment and the task are equally important parts of the system. Thus, the DSA’s starting point is that all components of the person, environment and task are equally important and could potentially contribute to the emerging behavior (cf. Newell, 1986).

According to the DSA, the components of the body-environment-task system are interacting. The result of the interaction at any point in time is the system’s current behavior. Hence, if one or multiple components change, the behavior might change. Thus, developmental trends emerge from changes in interactions that are affected by all components of the system. In contrast with ‘single-cause approaches,’ the DSA does not search for causal factors in development, but aims to reveal processes according to which behavior emerges from various contributing components. It also means that the component(s) involved in the emergence of new behavior may differ at each instant in development.

The concept that DSA uses to explain the emergence of new behavior is that of an attractor. Attractors are preferred, but not fixed, behaviors of the system to which the system returns to when perturbed. Attractors emerge from the interaction of the components at a certain point in time. At a given moment more behavioral attractors are present, hence, the attractor landscape represents the dynamic regime and the stability of the attractors emerging from interactions among task, person and environment components. Changes in the attractor landscape (reflecting disappearing behaviors, appearing behaviors, and qualitatively changing behaviors) are indicated in terms of stability and its counterpart variability. Stability of the attractor specifies resistance to change which is indicated by the effort it takes the system to perform a new or a different behavior. Weak attractor stability can result in an easy transition to a different attractor, which is reflected in increased behavioral variability. For development this means that when components of the system change, the interaction changes, which might influence the stability of the attractors in the attractor landscape. This changed attractor landscape can lead to different behavioral patterns becoming stable resulting in changes at the performance level, affecting development.

## Applying the Principles of DSA to Mid-Childhood Reaching

To understand developmental changes in reaching, the attractor landscape of reaching has to be identified (e.g., Schöner, 1990, 1994; Huys et al., 2014; Knips et al., 2017). Following Schöner (1990, 1994), we suggest that discrete reaching movements can be conceptualized as sequentially stabilizing point attractors (i.e., representing the initial and target location) and limit-cycle attractors (i.e., representing the movement) within a single dynamic system. The reaching movement is engendered by an intentionally destabilizing point attractor of the initial location while the limit cycle concurrently stabilizes (representing the actual displacement of the limb), followed by a destabilization of this limit cycle and subsequent relaxation toward the target location attractor. How do changes in the attractors of reaching lead to different patterns of change at the performance level?

The limit cycle in particular has effects on the performance of the reach because it accounts for the trajectory stability (Beek and Beek, 1988; Mottet and Bootsma, 1999; Zaal et al., 1999; Bongers et al., 2009). For instance, Mottet and Bootsma (1999) showed that to meet different task constraints imposed by modifications in target size and distance in a rhythmic reaching task, limit cycle dynamics systematically varied over conditions. In each condition, the imposed task constraints instantiated a limit cycle attractor of which the characteristics emerged from the interaction of the components involved in the system (target properties and person constraints). The exact dynamics of the limit cycle in turn determined the performance of the reaching movement.

### How to Explain Developmental Changes with the DSA

As described in the introduction the components that contribute to reaching are undergoing developmental changes during mid-childhood (i.e., joint coordination). All these changes in individual components affect the interaction, which changes the attractor landscape of reaching, i.e., the point attractors and the limit cycle attractor, resulting in changes in reaching behavior at different timepoints in development. Thus, 6-year-old children have different attractors than 8- and 10-year-old children which influences the performance of the reach in different ways, resulting in different movement speeds or accuracy scores at different ages.

### How to Explain the Different Shapes of Developmental Trends Revealed

As described earlier, different developmental trends were revealed in different studies. We gave the example of the studies of Bard et al. (1990) and Fayt et al. (1993) which both used similar age-groups, manipulated vision availability and focused on amplitude and directional aspects, but they found different results. Important for understanding that DSA can explain these changes is that the details of the experimental setups in these studies differed. For example, differences can be noticed in the task setup (moving a stylus on a tablet vs. reaching with a lever attached to the ground), the reaching distance (15 cm vs. 30 cm) and the target locations (10°, 20°, 30° of eccentricity to the right of the sagittal plane vs. 0°, 20°, and 40° of eccentricity). Such differences in setup affect the interactions between the components differently in each experiment, resulting in different attractor landscapes and therewith in different performance of the reach that produced different developmental trends for the different studies.

### From the Foregoing, a Perspective for Future Studies Emerges

The next step after presenting this promising approach in development is testing its ideas. Comparing developmental dynamics of different components or levels of analysis, such as the joint angle level and the performance level, can test the assumption that every component develops on its own timescale, which could for example be done with the Uncontrolled Manifold method (Scholz and Schöner, 1999; Latash et al., 2007). Related to this, explaining all findings of the literature regarding the effect of task and environment on dynamics of the reach, requires an encompassing dynamical model as a level-overarching account (cf. Thelen et al., 2001). Another important future focus should be on how changes in components affect attractors, and how this results in the specific performance found at a particular age.

## What Kind of Implications Does the Study of Mid-Childhood Reaching Have for Other Fields of Development?

Here, we have set out to provide a perspective that can offer an overarching explanation for a research field; mid-childhood reaching. The advantage of the DSA is that this perspective’s line of reasoning can be applied to other developmental fields because it is about general principles of change. We have already shown how these principles can also be applied to the behavior itself (i.e., the attractor landscape of reaching). These general principles provide a framework that over-arches individual studies and fields of studies. Other fields of development (e.g., language development) can therefore benefit from insights in, for example, the field of reaching. One important point that should be considered in all fields is that the effects of the context in which behavior is performed and the influence of individual characteristics should be determined. Here, we have argued that differences in experimental setups together with differences in developing components involved in reaching may explain different developmental trends. Therefore, in all fields of developmental research inter- and intra-individual variability should be more emphasized (cf. Adolph et al., 2015). Focusing on individual differences implies an experience driven-approach, as opposed to the often-used age-driven approach (e.g., which is also used here to follow the literature). Changes in intra-individual variability could indicate transitions to new behavior (i.e., changes in the attractor landscape). Also, variability may imply exploration which is valuable to understand how new behavior emerges. To conclude, with the example of mid-childhood reaching we have shown that the DSA can increase the understanding of emerging developmental changes.

## Author Contributions

Following authors contributed to the following aspects of the paper: (1) establishing the goal and main research question (LG, RB, MS, LM, EO); (2) reviewing the literature (LG, MS, RB); (3) drafting the work (LG, RB, MS); (4) establishing the core conceptional aspects (LG, RB); (5) critical revising of the work (LG, RB, MS, LM, EO). All authors gave final approval of the version to be published.

## Conflict of Interest Statement

The authors declare that the research was conducted in the absence of any commercial or financial relationships that could be construed as a potential conflict of interest.
